# Comprehensive approaches for diagnosis, monitoring and treatment of chronic inflammatory demyelinating polyneuropathy

**DOI:** 10.1186/s42466-020-00088-8

**Published:** 2020-12-08

**Authors:** Anna Lena Fisse, Jeremias Motte, Thomas Grüter, Melissa Sgodzai, Kalliopi Pitarokoili, Ralf Gold

**Affiliations:** 1 Department of Neurology, St. Josef-Hospital, Ruhr-University Bochum, Gudrunstrasse 56, 44791 Bochum, Germany; 2 Immunmediated Neuropathies Biobank (INHIBIT), Ruhr-University Bochum, Bochum, Germany

**Keywords:** Chronic inflammatory demyelinating polyneuropathy, Inflammatory neuropathies, Imaging, Pathophysiology, Diagnosis, Monitoring, Treatment, Register, Biobank

## Abstract

Chronic inflammatory demyelinating polyradiculoneuropathy (CIDP) is the most common chronic inflammatory neuropathy. CIDP is diagnosed according to the European Federation of Neurological Societies/Peripheral Nerve Society (EFNS/PNS) criteria, which combine clinical features with the electrophysiological evidence of demyelination. However, firstly, diagnosis is challenging, as some patients e.g. with severe early axonal damage do not fulfil the criteria. Secondly, objective and reliable tools to monitor the disease course are lacking. Thirdly, about 25% of CIDP patients do not respond to evidence-based first-line therapy. Recognition of these patients is difficult and treatment beyond first-line therapy is based on observational studies and case series only. Individualized immunomodulatory treatment does not exist due to the lack of understanding of essential aspects of the underlying pathophysiology.

Novel diagnostic imaging techniques and molecular approaches can help to solve these problems but do not find enough implementation. This review gives a comprehensive overview of novel diagnostic techniques and monitoring approaches for CIDP and how these can lead to individualized treatment and better understanding of pathophysiology.

## Background

The chronic inflammatory demyelinating polyradiculoneuropathy (CIDP) is the most common chronic inflammatory neuropathy. Chronic and recurrent polyneuritis was first described in 1890 by Eichhorst (Eichhorst H.: Polyneuritis recurrens. Correspondenzblatt f. Schweizer Ärzte 1890, publication not digitally available). Around 1950 reports about steroid responsive chronic polyneuritis arose. The term ‘chronic inflammatory demyelinating polyradiculoneuropathy’ was firstly described by Dyck et al. 1982 [1]. CIDP is a relapsing-remitting or progressive inflammatory neuropathy with a multifaceted presentation. There are multiple other chronic inflammatory neuropathies besides CIDP. In the past decades several diagnostic criteria for diagnosis of CIDP were established. The European Federation of Neurological Societies/Peripheral Nerve Society (EFNS/PNS) criteria [2] published in 2006 and revised in 2010, were validated in a multicenter European cohort and have since been broadly adopted in special for clinical trials. They combine clinical features with the electrophysiological evidence of demyelination. Despite these criteria misdiagnosis of CIDP is a problem.

About 25% of CIDP patients do not respond to evidence-based first-line therapy with steroids, plasma exchange and intravenous immunoglobulins. Individualized immunomodulatory treatment does not exist due to the lack of understanding of essential aspects of the underlying pathophysiology. Definition of treatment response is often difficult, as objective and reliable tools to monitor the disease course are lacking. This review gives a comprehensive overview of diagnosis, monitoring and treatment as well as pathophysiology of CIDP.

### The challenge of correct diagnosis and lucid terminology

Prevalence of CIDP is estimated between 0.8 to 8.9 cases per 100,000 [3–5]. Typically, more men than women are affected (2:1), and mean age is about 40–50 years [3–5]. Regional differences of prevalence between continents as known for acute inflammatory demyelinating polyneuropathies are not known for CIDP, as systematic data on epidemiology are lacking [6]. Dietary habits and antecedent infections may have an impact on the risk, onset and clinical presentation of the disease [7].

The challenge of the correct diagnosis is depicted through the fact that more than 15 sets of diagnostic criteria were used over the last 50 years [8, 9]. The currently most widely accepted criteria, the EFNS/PNS criteria, were established in 2005, revised in 2010 [2]. They combine clinical criteria with electrophysiological evidence of demyelination, while the evidence of inflammation is only included in the supportive criteria through consideration of nerve biopsy and magnetic resonance imaging (MRI). Clinically, EFNS/PNS criteria differentiate typical CIDP with proximal and distal weakness and sensory dysfunction of all extremities from atypical CIDP, in which predominantly distal, asymmetric or focal, pure sensory or pure motor symptoms occur. Supportive criteria also include elevation of protein in cerebrospinal fluid (CSF), response to treatment and abnormal sensory electrophysiology in at least one nerve. Additionally, laboratory exclusion of other conditions is demanded for correct diagnosis.

However, there are some limitations of EFNS/PNS criteria described below. Electrophysiological criteria are complex and extensive and therefore difficult to use in daily clinical practice. The use of incomplete electrophysiological protocols can lead to misdiagnoses and delayed diagnoses [9]. Also, clinical experience reveals patients i.e. with predominant axonal damage who do not fulfil electrophysiological criteria, although they probably have an inflammatory neuropathy.

The supportive criteria include some further difficulties as well. Breiner et al. suggested age-dependent cut-off values [10] for elevation of protein in CSF with a sensitivity of 80–90% and specificity of 50–60%, but the optimal cut-off value to avoid overdiagnosis is unclear [9]. Also, the role and right timepoint of nerve biopsy in detection of inflammatory infiltrates and demyelination compared to electrophysiological studies remains unknown [11–13]. MRI is difficult to use in everyday practice, due to the required technical expertise in specific imaging protocols and costs. Treatment response as a supportive criterion is not defined and challenging to objectify, possibly leading to over-diagnosis. Moreover, knowledge about pathophysiology of distinct subgroups like nodo- and paranodopathies is not yet represented in EFNS/PNS criteria.

Studies on somatosensory evoked potentials (SEP) to detect demyelination in CIDP showed that SEP are an useful additional tool to NCS [14]. Therefore, SEP are part of the EFNS/PNS additional criteria. However, in daily clinical practice, SEP may be time consuming and technically difficult to analyze and therefore do not play a major role.

Clinical definition of atypical CIDP mentioned in EFNS/PNS criteria is vague. 2018 Doneddu et al. [15] defined more specific and detailed criteria for atypical CIDP. Described subtypes are distal acquired demyelinating symmetric neuropathy (DADS) without proximal limb-trunk-face involvement, pure sensory CIDP without weakness and Lewis-Sumner syndrome with a multifocal distribution of symptoms, also called multifocal acquired demyelinating sensory and motor neuropathy (MADSAM). Again, these definitions are based on the clinical and electrophysiological aspects only and do not consider pathophysiology or novel imaging techniques. Definitions of Doneddu et al. partly seem to be somewhat rigid as patients may change from one clinical subtype to another during their disease course. A more precise characterization of atypical CIDP, both clinically and paraclinically, as well as consensus criteria for atypical CIDP are still lacking. As a result, misdiagnosis of CIDP is common, especially in patients that are classified as atypical CIDP.

**Fig. 1:**
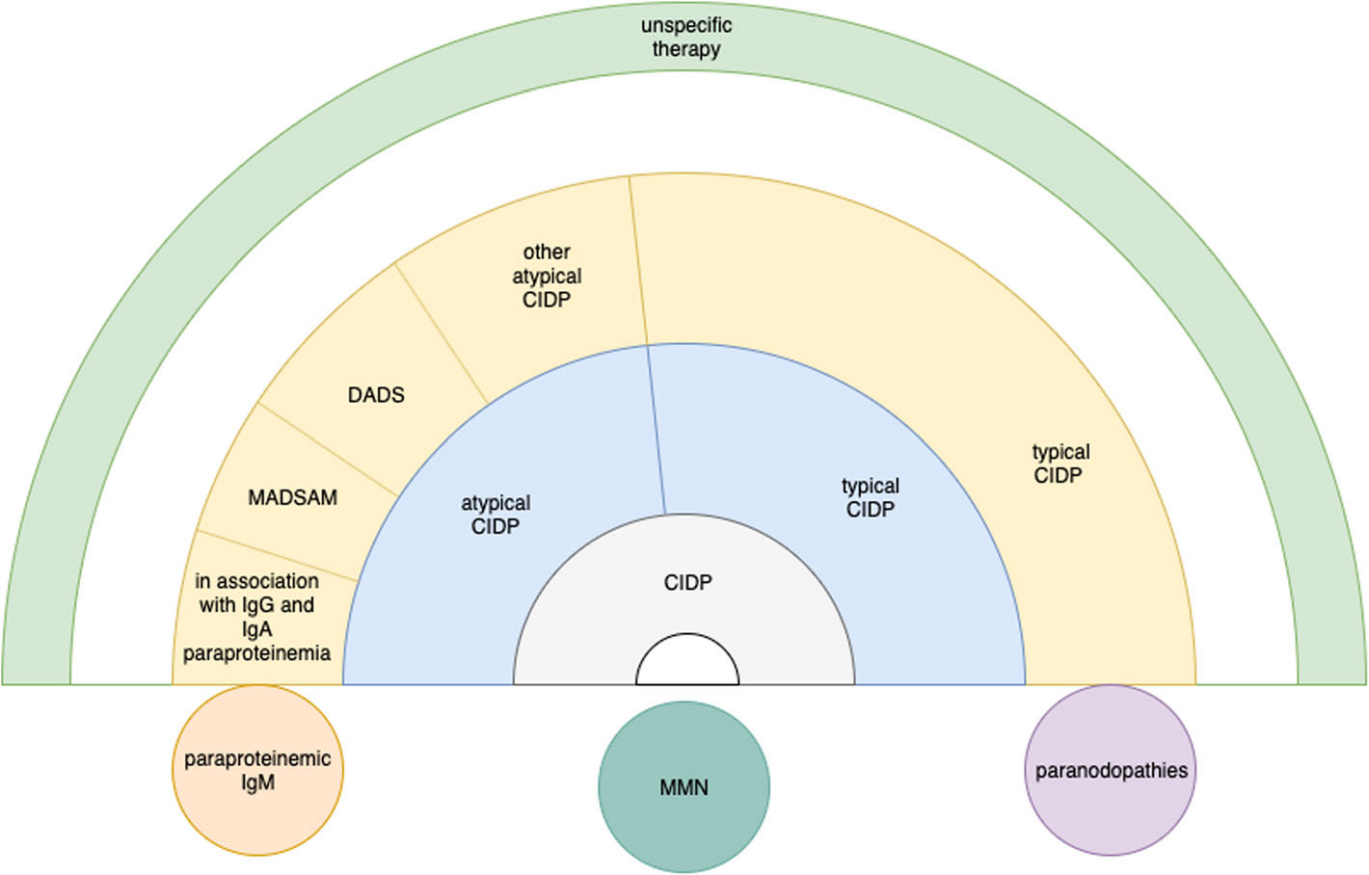
Overview of inflammatory neuropathies with focus on CIDP, subtypes and distinct disease entities

There are multiple other chronic inflammatory neuropathies besides CIDP with distinct pathophysiology such as multifocal motor neuropathy (MMN), paraproteinemic demyelinating neuropathies (PDN) with and without anti-MAG (Myelin-associated glycoprotein) antibodies as well as nodo- and paranodopathies. Further entities like MADSAM and DADS are defined as subgroups of CIDP but also have distinct clinical characteristics, treatment response and probably distinct pathophysiology. As the terminology of CIDP subgroups therefore seems to be heterogenous, the term ‘chronic inflammatory neuropathies’ (CIN) has been used in order to summarize all these entities [6] (Fig. 1). On the other hand, differentiation of subgroups and not lumping all entities together is necessary to enable individualized treatment and better understanding of pathophysiology.

### The challenge of clinical and electrophysiological monitoring

Classic methods for monitoring CIDP are clinical course and electrophysiology. Disability and symptom scores enable precise clinical characterization and comparison of symptoms in disease course in an objective manner.

- The Medical Research Council (MRC) sum score, originally developed for Guillain-Barré-Syndrome (GBS) patients in the 1970ies, is part of the standard repertoire of clinical examinations used to record muscle strength [16].
- The INCAT-Overall Disability Sum Score (ODSS), first described in 2002, is well-established and validated for patients with CIDP and has developed into a standard score for CIDP [17]. Yet, this score poorly detects discrete changes of disability or sensory symptoms.
- The INCAT sensory sumscore (ISS) is one of the few scores that sensitively records sensory symptoms in patients with GBS and CIDP [18].
- The Rasch-built Overall Disability Scale (R-ODS) is an improved disability score validated for CIDP, GBS and polyneuropathy associated with monoclonal gammopathy of unclear significance (MGUS). Indeed, it enables detection of minor changes compared to the INCAT-ODSS [19, 20].

In the recent years, symptoms other than sensorimotor impairment like quality of life [21] were in focus. Further symptoms like pain and fatigue need to be addressed in future studies.

A bedside tool to monitor grip strength is the Martin Vigorimeter which was shown to be a reliable and responsive tool in CIDP patients [22].

It appears obvious that a worsening of the disease can be depicted by nerve conduction studies. However, nerve conduction studies cannot reproduce clinical dynamics, i.e. due to severe secondary axonal damage [23]. Studies on use of electromyography for disease monitoring are lacking. Recently we described that evidence of persistent spontaneous denervation activity could display disease activity (own work under review). Yet, electromyography is invasive, painful and contraindications may prevent regular use. Therefore, use of electrophysiology for monitoring of CIDP is limited although there is extensive knowledge for many generations of clinical neurologists.

### Peripheral nerve and muscle imaging as novel diagnostic approaches

#### MRI

Morphologic alterations of nerves can be detected by MRI. Its main advantages are high-resolution and ability to image deep and proximal tissues. However, differences in acquisition and analysis may result in significant limitations regarding validity. Short tau inversion recovery (STIR) sequences and nerve-specific T2-weighted magnetic resonance neurography (MRN) are used to quantify hypertrophy and depict increased signal intensity as signs of inflammation. Diffusion tensor imaging (DTI) enables evaluation of microstructural integrity using the parameter of fractional anisotropy, which indicates demyelination [24, 25]. However, these novel techniques are only used in selected patients or as part of cross-sectional studies. Broadly available MRI rather enables imaging of spinal roots and brachial and lumbosacral plexus to depict hypertrophy and gadolinium-enhancement which is represented as supportive criterion in EFNS/PNS criteria.

#### Nerve ultrasound

**Fig. 2:**
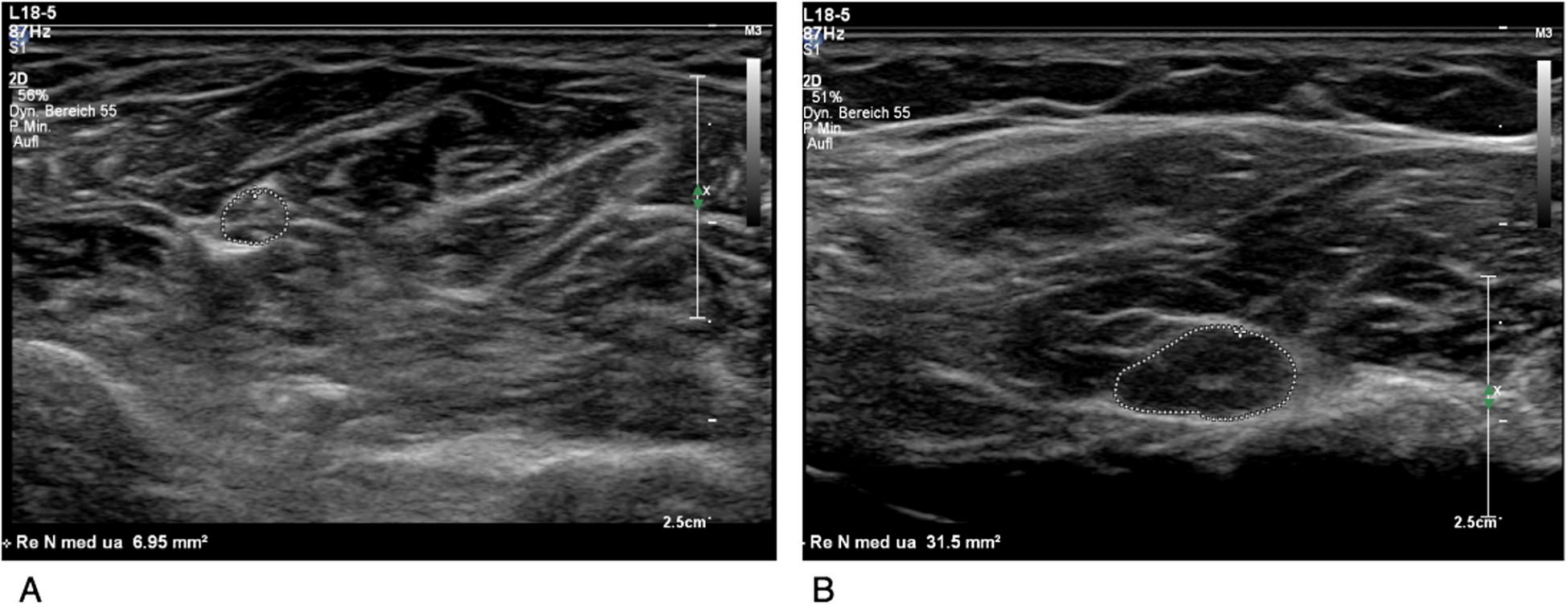
**a** Normal median nerve at the middle of the forearm between the flexor digitorum profundus and superficialis muscles with a normal CSA of 6.95 mm^2^ and normal fascicular structure. **b** Significantly enlarged median nerve at the forearm with a CSA of 31.5 mm^2^ in a patient with CIDP. Some swollen fascicles and a hypoechoic structure can be depicted. Ultrasound stetting except focus are the same in both images

Nerve ultrasound also enables a non-invasive view of morphology of affected peripheral nerves. The benefit of nerve ultrasound in the diagnosis of CIDP has been proven several times over the past 7 years, but it is still not established as standard diagnostic criterion. The measurement of cross-sectional area (CSA) in ultrasound correlates well to CSA detected by MRI and with the nerve T2-weighted signal intensity [26–28]. Morphological changes like swollen, hypoechogenic nerve and fascicles detected in ultrasound represent acute inflammation [29, 30], while hyperechogenic nerves rather are supposed to occur in case of fibroid remodeling and axonal damage [29, 31]. Thus, measurement of CSA on the one hand, and assessment of the echogenicity on the other hand are the main parameters for assessing CIDP by ultrasound. Figure 2 exemplarily shows an ultrasound image of a normal median nerve at the forearm of a healthy person and a significantly enlarged nerve of a CIDP patient.

CSA enlargements can occur focally, multifocally or more generalized. Frequently, not only enlarged CSA of the whole nerve, but also individual enlarged fascicles can be observed in some CIDP patients. Multiple publications have shown the value of HRUS as an add-on tool to electrophysiological examinations for the diagnosis of CIDP and several ultrasound protocols, normal values and scores based on CSA were published to diagnose CIDP and differentiate it from GBS as well as differentiation protocols for atypical CIDP forms [32–35]. Published nerve ultrasound protocols distinguish acute and chronic inflammatory polyneuropathies as well as hereditary polyneuropathies [33, 36]. It has not yet been investigated, whether nerve ultrasound also helps to distinguish axonal non-inflammatory polyneuropathies from CIDP with secondary axonal damage.

The correlation between a morphologically focal swollen nerve observed by ultrasound and a corresponding clinical and electrophysiological damage is still under discussion [37]. In other diseases such as entrapment syndromes or pressure palsies, the morphological change often correlates with the function [38]. For example, in cases of acute pressure palsy of radial nerve, conduction blocks can often be found at exactly that section of the upper arm where sonomorphologically focal CSA enlargement occurs [39]. For CSA enlargement in CIDP, only some authors have described similar connections [37, 40]. It was suggested that inflammatory morphological changes in CIDP can probably be displayed by ultrasound even before functional and electrophysiological impairment.

In disease course, increase or decrease of CSA enlargement, i.e. measured by intra-nerve CSA variability, can provide information about disease activity and response to therapy [23, 41].

Regarding evaluation of echogenicity, HRUS might be useful as prognostic tool, as it was shown that patients with hyperechogenic nerves have a worse prognosis than that with hypoechogenic nerves. Also, the extent of hypoechogenic fraction often occurring along with CSA enlargement correlates to disease course [31, 42].

A limitation of nerve ultrasound is that proximal and deep nerves such as lumbosacral plexus cannot be displayed and that the quality of imaging is dependent on the expertise and experience of the examiner.

#### Muscle ultrasound

Muscle ultrasound in CIDP was described to be useful to detect secondary axonal damage via reduced muscle thickness and hyperechogenic remodeling of muscles in one CIDP study [43]. In conditions like motor neuron diseases, muscle ultrasound can also be used to detect fasciculations [44–46], even better than electromyography [47].

#### Corneal confocal microscopy

**Fig. 3:**
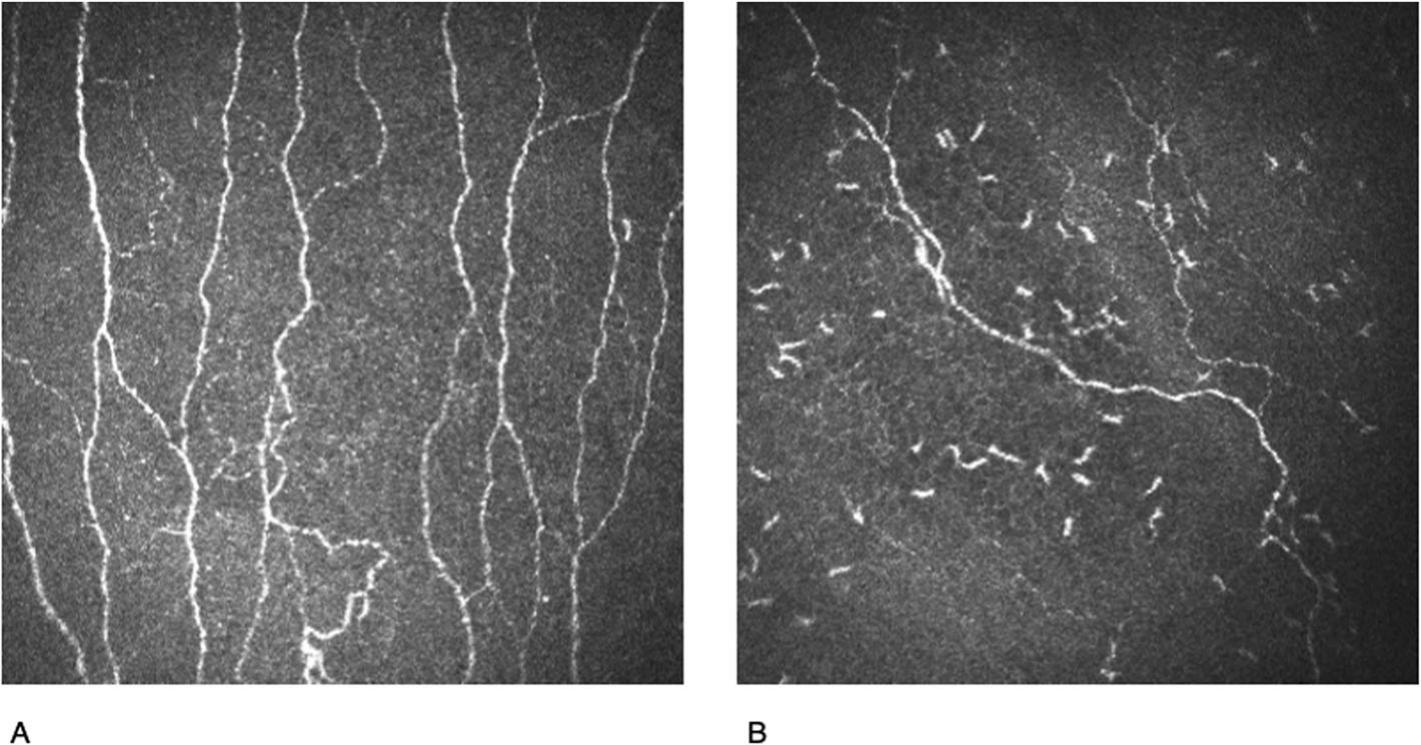
Corneal confocal microscopy showing nerve fiber reduction and immune cell infiltration in a CIDP patient with progressive disease course (**b**) compared to a healthy person (**a**). (With the kind permission of Professor Martin Tegenthoff, Bochum, and Dr. Dietrich Sturm, Wuppertal, 2020)

Corneal confocal microscopy (CCM) is a novel promising tool for evaluation of disease activity in CIDP. As a transparent medium, the cornea allows nerves of the subbasal plexus of the ophthalmic branch of the trigeminal nerve and immune cells to be visualized in vivo [48, 49] and opens up possibilities for the detection of nerve fiber reduction (Fig. 3). For inflammatory-demyelinating diseases such as CIDP, there are contradictory results about the diagnostic value of the nerve fiber length, density and branching shown by CCM [50, 51]. It is currently still unclear whether these parameters change dynamically in the course of the disease. Moreover, corneal nerve fibers are surrounded by immune cell populations, which can easily be quantified and change dynamically during disease course and might correlate with disease activity [28].

#### Conclusion of the novel strategies in diagnosis and monitoring: what could be improved based on current knowledge?

The EFNS/PNS criteria were developed for use in daily clinical care as well as in clinical trials. Nevertheless, they are too complicated for routine use in non-specialized centers and misdiagnosis of CIDP is a current problem. MRI is the only imaging technique mentioned as supportive criterion in EFNS/PNS criteria that involves a high level of technical effort. In contrast, ultrasound is widely available, relatively easy to learn and efficient but is not yet included in the criteria. Nerve imaging offers the possibility of directly imaging inflammation. Nerve ultrasound should play a greater role in diagnosis of CIDP in the future as part of the diagnostic criteria, complementary to electrophysiology and MRI. As monitoring tools nerve conduction studies have shortcomings and the relevance of electromyography is not examined sufficiently. Neuromuscular ultrasound and CCM are promising approaches for disease monitoring, but the number of studies is too small to generally recommend them as standard diagnostics and monitoring. Hence, there is currently no solitary technical method that can reliably track the course of the disease.

### The challenge of understanding nerve inflammation in CIDP

The clinical heterogeneity, different diagnostic patterns and different treatment response suggest distinct immunological pathophysiology in CIN, CIDP and subgroups. Humoral and cellular, T-cell driven, autoantibody-induced as well as complement-mediated autoimmunity occurs in all CIN and synergistically lead to damage of peripheral nerves [52].

A crucial step is the break-down of the blood-nerve barrier, indicated by increased protein in CSF, gadolinium enhancement in of the nerve trunks, roots and plexuses in MRI studies as well as nerve swelling in ultrasound studies [52–54]. Activated CD4^+^ T-cells play a major role here, as they are the first cells to cross the blood-nerve barrier. The migrating T-cells secrete cytokines and chemokines and enable macrophages and antibodies to enter the peripheral nervous system. Different CD4^+^ T-cell subsets (Th1, Th17) were described in CIDP subgroups, which indicate differences in underlying T-cell responses between atypical and typical CIDP [55]. Sural nerve biopsies show CD4^+^, CD8^+^ cells and macrophages [56–59], but also immunoglobulin and complement on the outer surface of Schwann cells and the compact myelin [60, 61].

The concept of classical macrophage-induced myelin destruction, reduced conduction velocities and conduction block resulting from segmental demyelination is considered typical of CIDP and related disorders [62, 63].

Indeed, macrophages are the key player in this concept. They appear as antigen-presenting cells, create a pro-inflammatory environment, destroy the myelin through phagocytosis and directly attack the myelin [64, 65]. This results in early secondary axonal damage in CIDP [66, 67].

Nerve biopsy shows features of segmental demyelination and remyelination, onion bulb formation (as a result of repeated thin-regenerating and demyelinating Schwann cell effort), nerve edema and occasionally T-cells [68]. Inflammatory infiltrates are typically both endoneurial and epineurial and frequently perivascular [69]. Macrophages are scattered either throughout the endoneurium or in small perivascular clusters in the endoneurium and mediate demyelination [69–71]. Demyelination typically occurs paranodally and only for a short time, Schwann cells quickly remyelinate the destroyed segment insufficiently with shorter internodes and thinner myelin resulting in the onion bulb formation. Schwann cells upregulate the antigen presenting major histocompatibility complex class (MHC)-II, so that a pro-inflammatory environment is maintained in the nerve.

Increased systemic concentrations of TNFα and Interleukin-2 are markers for T-cell activation [72, 73]. However, a single triggering antigen has not yet been found. Therefore, a strong evidence for a molecular mimicry-like mechanism as presumed in GBS is absent. Antibody responses against the myelin proteins P0 and P2 [74] and peripheral myelin protein 22 (PMP22) have been reported [75, 76] but are under discussion [77, 78]. The detection of T cells with γδ-receptors in nerve samples of CIDP underlines the possible pathogenetic role of cellular immune response against non-protein antigens like gangliosides [79].

There is increasing evidence that MHC-I-restricted, CD8^+^ T-cell-mediated attack against peripheral nerve tissue components contributes to the pathogenesis of CIDP [56, 80, 81]. The role of CD8^+^ T-cells is controversial. Similar clonal expansion of CD8^+^ cells in sural nerve biopsies and peripheral blood was described [56]. Also, the analysis of the T-cell repertoire in peripheral blood of CIDP patients showed an extensive oligoclonal expansion in CD8^+^ T-cells that was reduced after treatment with intravenous immunoglobulins (IVIg) [82]. An increase of natural killer-T-cells and CD8^+^ T-cells in CSF of CIDP patients was shown recently [81]. These studies suggest a central role of cytotoxic cell types in inflammatory neuropathies. However, up to now, no foreign- or self-antigen has been identified as a CD8^+^ target in CIDP. Also, in experimental autoimmune neuritis (EAN, animal model of CIDP), CD8^+^ T-cells do not play a significant role [52]. However, EAN may represent human CIDP insufficiently and animal models driven by CD8^+^ T-cells have to be developed. Also, it is currently unknown whether CD8^+^ T-cells mediated pathophysiology is part of classical macrophage-induced myelin destruction or a distinct entity.

The role of B-cells is incompletely understood. In spontaneous autoimmune polyneuropathy (SAP) in non-obese diabetic mice, depletion of B-cells and plasmablasts with anti-CD19 antibodies leads to the prevention and attenuation of SAP [83]. The role of regulatory B-cells was described as secondary in this model [84]. In contrast, in immune cell profiling of CSF in CIDP a B-cell pattern was not found [81].

Yet, therapy response after using rituximab as a CD20-depleting antibody in CIDP patients may imply a relevant role of B-cells unless their antigen-presenting capacity toward T-lymphocytes stands in the center.

The efficacy of plasma exchange [85] and bortezomib depleting plasma cells [86] in CIDP indicates a pivotal role of humoral mechanisms. Also, immunoglobulin and complement were found deposited on the outer surface of Schwann cells and the compact myelin in sural nerve biopsies [60, 61]. They were also detected in sera and CSF from CIDP patients [87, 88]. Activation of complement system is considered as a relevant part of CIDP pathophysiology [65].

In the context of macrophage-induced demyelination an antibody-mediated pathway is assumed [65]. Autoantibodies from the serum of CIDP patients directed against various myelin proteins such as P0, P2, PMP22 were reported to trigger demyelination after passive transfer in the animal model [52, 87]. However, other authors did not confirm these findings [6, 75, 89]. Therefore, the role of autoantibodies directed against compact myelin proteins in CIDP is still unclear [90].

In contrast, the identification of antibodies against nodal and paranodal structures brought significant progress in understanding about the role of autoantibodies in immune neuropathies [91–93]. The nodal antibodies target antigens such as the nodal proteins neurofascin (NF) 186 and gliomedin or paranodal proteins as Contactin-associated protein (CASPR) 1, NF 155 and contactin 1. These proteins are important in clustering Na^+^-channels and to maintain the functional structure (“compartmentalization”) of the myelinated axon essential for the saltatory conduction. The paranodal disarrangement resulting from the attachment of IgG4 at paranodal junctions and the absence of macrophage-induced demyelination are characteristic pathologic features in patients who have these antibodies [94, 95]. Antibodies against NF 155 and contactin 1 are of IgG4 class, which are not complement-activating [65]. These mechanisms are different to demyelination in classical macrophage-induced CIDP, which is why paranodopathies should be regarded as a distinct disease entity.

The incidence of these paranodal antibodies is reported around 2–13% in CIDP patients [52, 91, 92]. Some authors recommended the term “seropositive CIDP” for these patients [96]. Patients are clinically characterized as younger than typical CIDP, with a subacute and more severe onset, disabling tremor, ataxia, distal dominant weakness, and poor response to IVIg [96]. It is not known why these (para-) nodal proteins become an autoimmune target. Moreover, unknown nodal autoantibodies are reported in up to 30% of CIDP patients [97], showing the need for differentiation of further antibodies.

### The challenge of understanding aetiology of CIDP

#### Nutrition and environmental factors

The data on the influence of nutrition and environmental factors in CIDP are scarce. Several studies have shown an association of diet and environmental factors like tobacco and alcohol consumption with the progression of disability in different autoimmune diseases [98, 99]. In neuroinflammatory and neurodegenerative diseases, the immunological influence of the gut microbiome has increasingly gotten into focus of research [100]. Short-chain fatty acids are reduced in autoimmune diseases as a consequence of an altered gut microbiome. This leads to reduced regulatory T-cell function. Supplementation of short-chain fatty acids like propionic acid shows beneficial immunomodulatory effects, as well as a neuroprotective effect in multiple sclerosis [101, 102]. So far for diseases of the peripheral nervous system short-chain fatty acids were only described in a single case report [103].

Capsaicin is an alkaloid contained in spicy food. It is a direct agonist of the transient receptor potential channel vanilloid subfamily member 1, that is expressed in different cell types of the nervous system as well as the immune system. This receptor and its modification by capsaicin shows beneficial immunomodulatory effects in EAN [104] as well as in Schwann cells [105].

In real life the influence of these factors is under discussion. In a study with 323 CIDP patients physical activity improves symptom severity, disability and quality of life, but other environmental factors as smoking, alcohol and different dietary regimes did not have an impact on the severity and health perception of CIDP [98].

#### Genetics

The association between genetic risk factors and CIDP is under discussion and not yet clearly described. There have been many attempts to find a human leukocyte antigen (HLA) association with CIDP with little success [106]. Some genes show association with disease severity rather than disease susceptibility [106]. The greatest problem is the limited numbers of subjects in the genome association studies. Therefore, it would be beneficial to repeat the studies with larger cohorts [106] as there is need for more detailed molecular studies about HLA influence and modern genetic approaches to CIDP.

### The challenge to find potential biomarkers

Serum neurofilament light chain (sNfL) levels are increased in about a third of CIDP patients and seem to reflect ongoing axonal damage in the peripheral nervous system. sNfL could therefore be a potential biomarker of disease activity however further studies are needed [107–109].

Compound scores created from multi-dimensional CSF parameters like immune cell subtypes could become potential novel diagnostic tools as they differentiate distinct disease mechanisms in subgroups of inflammatory neuropathies [81].

#### What can we learn from current knowledge of pathophysiology, aetiology and biomarkers for clinical routine?

The pathophysiology of CIDP shows complex inflammatory mechanisms that result not only in demyelination but also in early axonal damage. Therefore, clinical classifications without regarding pathophysiology cannot be comprehensive. The discovery of nodo- and paranodopathies in the recent years illustrates how knowledge of distinct pathophysiology can lead to clinical differentiation of new disease entities. Therefore, biomaterial like blood, CSF and neural tissue of patients with atypical or unusual clinical characteristics needs to be available to the scientific community for further research.

This further understanding of the pathophysiology is crucial to enable specific treatment options.

### Treatment of CIDP

#### First line treatment options: immunoglobulins, corticosteroids (STE) and plasma exchange (PE)

##### Immunoglobulins

In ICE trial, the largest and longest randomized-controlled trial comparing intravenous immunoglobulin (IVIg) treatment to placebo in 117 patients for 24 weeks, IVIg were applied with an initial dosage of 2 g/kg body weight for 2–4 days, maintained by 1 g/kg body weight for 1–2 days every 3 weeks [110], with a therapy response of 54%. Dose reduction after stabilization is attempted individually in everyday clinical practice. Alternatively, subcutaneous application of immunoglobulins (SCIg) in a dosage of 0.2–0.4 g/kg per week with individual dosing after stabilization was shown to be effective in 172 patients compared to placebo. 65–81% of patients treated with SCIg were relapse free in this study [111]. Subcutaneous administration of immunoglobulins is a novel effective option for CIDP patients, enabling convenient treatment at home, reducing infusion-related side-effects and outpatient visits for CIDP patients.

Mechanism of action of immunoglobulins is multimodal. IgG as the major component of IVIg are considered responsible for most of the immune-modulating effects [112, 113]. Antigen-binding fragment (Fab)-dependent effects like blockade of cell-cell interactions and neutralization of cytokines, activated complement proteins and autoantibodies, as well as fragment crystallizable region (Fc)-dependent pathways like competitive blockade of low-affinity Fc-Receptors, modulation of Fc-Receptor expression, and saturation of the Fc-Receptors have been described [114, 115]. These effects ultimately result in a modification of inflammatory mediators, downstream signaling molecules of inflammatory cascades and changes in activation of immune cells [113, 114]. Difference of immunoglobulin levels in serum before and after IVIg treatment is considered as possible marker for therapy response, as CIDP patients with low post-treatment IgG levels resulting from fast decay of IVIg are associated with clinical worsening during IVIg treatment [116].

##### Corticosteroids

Regarding STE treatment a Cochrane Review from 2017 revealed low evidence levels for treatment with oral or intravenous STE, mostly based on observational, but not on randomized controlled studies [117]. Nevertheless, corticosteroids are commonly used in practice. In a retrospective trial, response rate to STE treatment was around 60% [118]. Recommended treatment regimens are prednisolone 1 mg/kg per day orally, or intermittent high dose therapy with 500-1000 mg methylprednisolone for 3–5 days.

##### Plasma exchange

For PE, several available small trials have shown that between 33 and 66% of CIDP patients have significant short-term improvement from PE, but rapid deterioration may occur after completion of treatment [85]. Reliable data on long-term effects of PE are not available. Usually 5–10 cycles of PE are applied during 2–4 weeks. Immunoadsorption was reported as equally effective as PE [119].

First-line therapies are non-specific and which of these first line therapies is suited best for which patient is still not understood. Future challenge is to differentiate further subgroups to enable more specific therapies.

##### Other immunotherapies

For CIDP patients who do not respond to first line therapies treatment is challenging due to lack of studies. About 25% of patients do not respond to any of the three first-line therapies [86, 120]. Further treatment options for these patients include immunosuppressants or even an autologous stem cell transplantion. However, sufficient data on therapy response from controlled trials do not exist and therapies can have severe side effects [121–123]. Randomized studies are only available for azathioprine [124], interferon beta-1a [124–126], fingolimod [127] and methotrexate [128] showing no significant treatment response, but these studies were not large enough to detect minor or moderate benefit [121].

Observational studies including cyclophosphamide, ciclosporin, mycophenolate, rituximab, bortezomib as proteasome antagonist and peripheral blood stem cell transplantation have been performed with positive results, but do not provide enough high-level evidence [121]. Two commonly used drugs are rituximab and cyclophosphamide. Sixty CIDP cases with a response rate of 78% to rituximab have been reported in different case series [121, 129–132]. For cyclophosphamide fifty-one CIDP cases have been reported, of which 35 (69%) benefited from therapy [121]. A novel increasingly used treatment in refractory CIDP is bortezomib, a proteasome inhibitor for which efficacy was described in ten CIDP patients [86].

The knowledge about distinct entities of CIN like PDN or nodo- and paranodopathies led to more specific therapies in these diseases:

PDN are defined as demyelinating neuropathy, clinically often with DADS phenotype, and proof of IgM-paraproteinemia with or without anti-MAG-antibodies [133]. PDN is known to respond poorly to standard CIDP therapy with IVIg, STE or PE. Also, effect of other immunosuppressants is limited [133, 134]. Some observational and small controlled studies with cumulatively over 200 PDN patients suggest that rituximab can be helpful in 30–50% of these patients [134, 135], probably by suppressing IgM and anti-MAG antibody production, however, large randomized controlled studies are lacking.

There are no prospective data about treatment of nodo- and paraponopathies, but observational studies show low therapy response to IVIg [136, 137]. A possible cause of poor IVIg response is that autoantibodies are often IgG4 subgroup, which is not complement activating [96, 137]. Good response to rituximab was reported in small case series [96, 138, 139].

##### Drug free treatment options

There is evidence from some studies with CIDP patients showing the effect of strength training and physiotherapy to improve muscle strength [140]. Further approaches to improve the quality of life are neurostimulation or electrostimulation, although no controlled studies are available on this in CIDP, but only in other polyneuropathies and central diseases [141, 142].

#### What can we conclude about the treatment of CIDP?

The first line therapy of CIDP is immunologically unspecific. In more than 20% of the patients these first-line fails. If a patient does not respond to first-line treatment reevaluation of the diagnosis is mandatory. The variable treatment response of different subtypes underlines the importance of improving our knowledge on pathophysiology. But even without specific knowledge of the pathophysiology of certain subtypes, the investigation of subgroup-specific treatment responses is needed to improve treatment response rates.

## Conclusion

CIDP and other chronic inflammatory neuropathies have been better understood in recent years. There are new molecular biological findings and innovative approaches to diagnose and monitor inflammatory neuropathies. Nevertheless, the translation of new diagnostic approaches and new subgroups into diagnostic criteria lacks. Moreover, there is a great need for further knowledge about pathophysiology and therapeutic options of ‘seronegative’ CIDP itself. Further characterization of distinct subgroups i.e. progressive or treatment refractory patients could enable targeted and more individualized therapy. Until now, treatment decisions in atypical and progressive CIDP are not evidence-based due to the lack of large controlled studies. We hope that the era of individualized treatment in CIDP will make significant progress.

Previous studies, regardless of whether they investigate pathophysiology, biomarkers, genetic or environmental associations or treatment of CIN, have in common that the high number of patients to achieve significant results is difficult to obtain. A further challenge along with the emerging knowledge in clinical and molecular research is to link all these aspects. Possible reasons for delayed implementation of the different fields in CIN are the high degree of specialization of the individual international working groups and inhomogeneous structure of data.

**Fig. 4:**
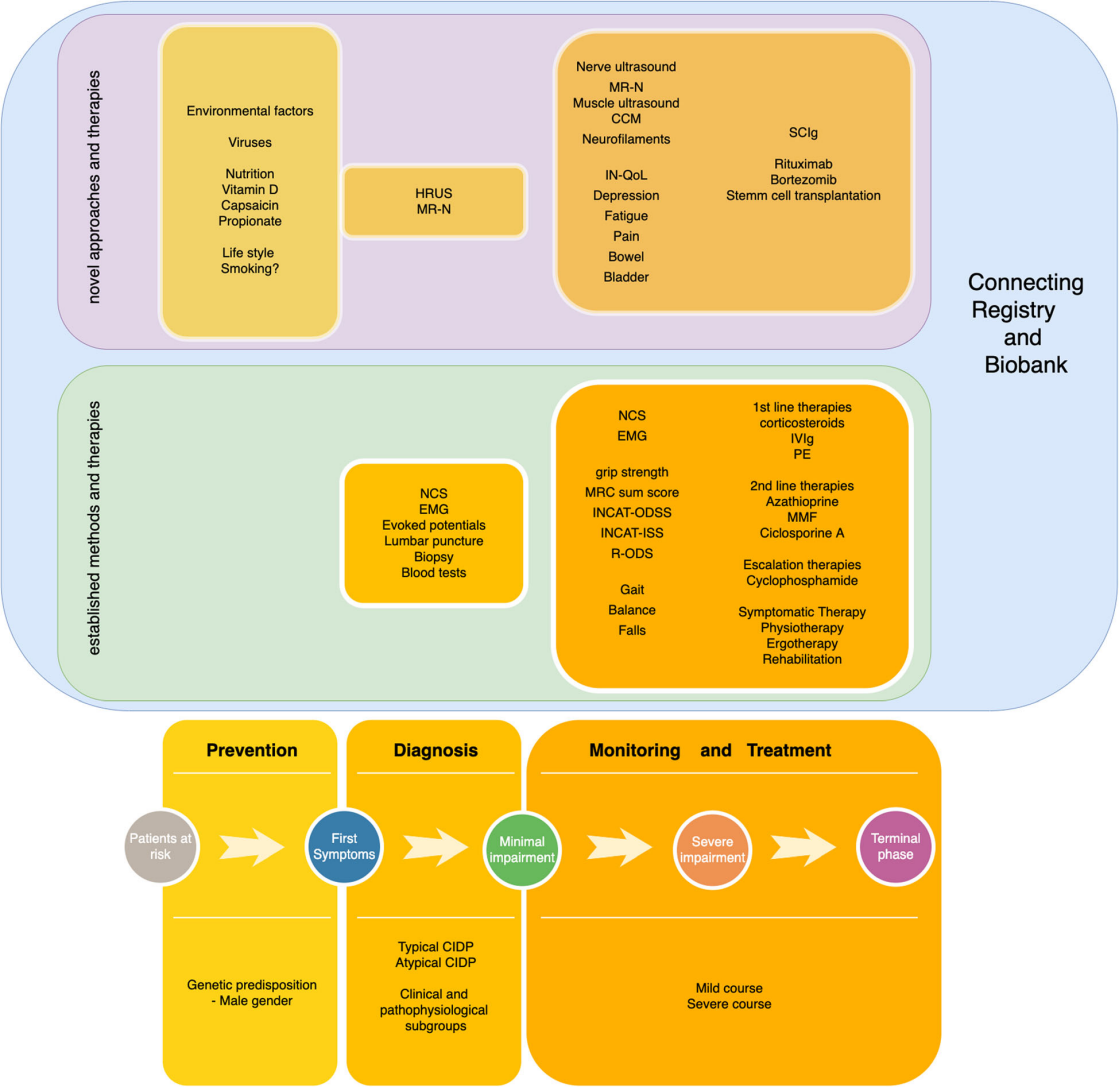
Overview of comprehensive approaches for diagnosis, monitoring and treatment of CIDP

Future research about CIDP with its subtypes and pathophysiology, as well as their therapy, requires combination of high-quality prospective clinical data with the opportunity to structured collection of biomaterials from large numbers of patients. This can only be achieved through multicenter, multidimensional registers for immune-mediated neuropathies with attached biobank (Fig. 4). A high degree of standardization with standard operation protocols must be obtained for biomaterials and their processing in order to make the results valid and comparable. All clinical characteristics, neuroimaging and molecular biological examinations and a comprehensive collection of biomaterials need to be sampled. The aim is the recognition of specific characteristics of the various subtypes of the disease. In addition to a better understanding of the disease, the development of targeted and individualized therapies will be a result of this work.

## Data Availability

Not applicable (review article).

## Abbreviations

CIDP: Chronic inflammatory demyelinating polyradiculoneuropathy
EFNS/PNS: European Federation of Neurological Societies/Peripheral Nerve Society
MRI: Magnetic resonance imaging
CSF: Cerebrospinal fluid
DADS: Distal acquired demyelinating symmetric neuropathy
MADSAM: Multifocal acquired demyelinating sensory and motor neuropathy
MMN: Multifocal motor neuropathy
PDN: Paraproteinemic demyelinating neuropathies
MAG: Myelin-associated glycoprotein
CIN: Chronic inflammatory neuropathies
MRC: Medical Research Council
GBS: Guillain-Barré-Syndrome
ODSS: INCAT-Overall Disability Sum Score
ISS: INCAT sensory sumscore
R-ODS: Rasch-built Overall Disability Scale
MGUS: Monoclonal gammopathy of unclear significance
STIR: Short tau inversion recovery
MRN: Magnetic resonance neurography
DTI: Diffusion tensor imaging
CSA: Cross-sectional area
CCM: Corneal confocal microscopy
MHC: Major histocompatibility complex class
PMP22: Peripheral myelin protein 22
IVIg: Intravenous immunoglobulins
EAN: Experimental autoimmune neuritis
SAP: Spontaneous autoimmune polyneuropathy
NF: Neurofascin
CASPR: Contactin-associated protein
HLA: Human leukocyte antigen
sNfL: Serum neurofilament light chain
STE: Corticosteroids
PE: Plasma exchange
SCIg: Subcutaneous immunoglobulins
Fab: Antigen-binding fragment
Fc: Fragment crystallizable region
